# Readability and Quality of Online Information on Osteochondral Knee Injuries: An Objective Assessment

**DOI:** 10.7759/cureus.85014

**Published:** 2025-05-29

**Authors:** Alexander Price, Xander Van Heerden, Samher Jassim, Fintan J Shannon

**Affiliations:** 1 Trauma and Orthopaedics, University Hospital Galway, Galway, IRL; 2 Trauma and Orthopaedics, University Hospital Waterford, Waterford, IRL; 3 Trauma and Orthopaedics, Tallaght University Hospital, Dublin, IRL

**Keywords:** health literacy, knee, osteochondral injuries, patient education, readability

## Abstract

Background

In the modern healthcare era, the internet serves as a major source of information for patients. However, prior studies have shown that online medical information frequently exceeds the recommended readability levels, limiting patient understanding. In the US, the average reading level is between seventh and eighth grade, while leading health organisations recommend that patient information not exceed a sixth-grade level. This study evaluates the readability and quality of publicly accessible online content related to osteochondral injuries of the knee.

Methods

A systematic search was conducted on Google (Google, Inc., Mountain View, CA), Bing (Microsoft® Corp., Redmond, WA), and Yahoo (Yahoo, Inc., New York, NY) using the terms “osteochondral defect knee” and “osteochondral injury knee.” The top 30 uniform resource locators (URLs), for each search term, from each search engine were screened. Readability of the content was assessed using four standardised readability metrics (Gunning Fog Index, Flesch-Kincaid Grade, Flesch Reading Ease, and Simple Measure of Gobbledygook (SMOG) index), while quality was measured based on the Journal of the American Medical Association (JAMA) benchmark criteria.

Results

Forty-six unique webpages were included in the analysis. The mean Flesch-Kincaid Grade Level was 10.1 ± 3.4, the mean Gunning Fog Index grade was 11.5 ± 4.1, the mean Flesch Reading Ease score was 43.8 ± 12.8, and the mean SMOG grade was 8.8 ± 2.9. Only five webpages were at or below a sixth-grade reading level. The mean JAMA score was 1.43 ± 1.46 out of four.

Conclusion

This study assessed the readability and credibility of online health information. The majority of online resources related to osteochondral knee injuries are difficult to read and lack key quality indicators. Improving both readability and reliability is essential to support patient comprehension, informed decision-making, and promote better health literacy.

## Introduction

Osteochondral injury repair, commonly referred to as knee cartilage repair surgery, is a common treatment for significant cartilage damage in the knee joint resulting from trauma or focal chondral defects, including specific cases of osteochondritis dissecans [1,2]. Techniques, including microfracture, osteochondral autograft transfer (OATS) and autologous chondrocyte implantation (ACI), are used to treat knee joint cartilage damage [3,4]. These procedures aim to restore joint function and reduce pain, improving quality of life and independence [5,6]. As patients increasingly seek information online, understanding the quality and accessibility of online digital resources becomes crucial.

The rapid expansion of digital health resources has led to an increasing number of patients seeking online information regarding procedural details, expected outcomes, and rehabilitation protocols [4]. As of January 2025, approximately 5.5 billion people globally use the internet, accounting for 69% of the global population, with over 1.38 billion active iPhone users [7]. While this enables broader access to health knowledge, it also introduces variability in the quality and complexity of available content. Incomplete or partial knowledge can often lead to misunderstandings and errors in judgment.

Despite efforts by health organisations to provide patient-centred online education, studies show many resources remain too difficult for the general population [8-10]. The US average reading level is seventh to eighth grade, yet materials often exceed this, conflicting with recommendations by the AMA and NIH that target sixth grade level [8-10]. This is particularly concerning for patients preparing for procedures like osteochondral repair, where realistic expectations and understanding of rehabilitation are critical.

This study aims to objectively evaluate the readability and quality of online resources related to osteochondral knee injuries, hypothesising that most will not meet the recommended standards.

## Materials and methods

Literature search

A systematic search was performed by two independent reviewers on March 16, 2025, using the three most popular search engines, these being, Google (Google, Inc., Mountain View, CA) (78.83%), Bing (Microsoft® Corp., Redmond, WA)(12.23%) and Yahoo (Yahoo, Inc., New York, NY) (3.07%), which accounts for approximately 94% of search engine usage across the market as of January 2025 [11]. The search terms "osteochondral defect knee" and "osteochondral injury knee" were applied to each search engine. The top 30 uniform resource locators (URLs), for each search term, from each platform, were collated, producing 180 URLs in total.

Inclusion criteria were English-language webpages and webpages containing written information, specifically discussing the topic of osteochondral knee defects.

Exclusion criteria included duplicate URLs, inaccessible or broken links, video-only webpages, and academic journal articles that required subscription or paywall access.

**Figure 1:**
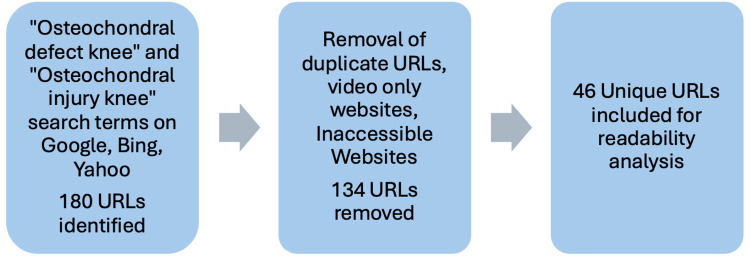
Flow diagram of literature search

After applying these criteria, a total of 46 unique URLs were included in the final analysis (Appendices). See Figure 1 for the literature search flow diagram.

Readability assessment

Readability was evaluated using the WebFX Digital Marketing (WebFX, Harrisburg, PA) readability online tool [12]. Each URL was entered into the tool, which generated scores for the Gunning Fog Index, Flesch-Kincaid Reading Grade Level (FKG), Flesch Reading Ease (FRE), and the Simple Measure of Gobbledygook (SMOG) index (Table 1) [13]. Where URLs did not yield results, the text was manually copied from the webpage and pasted into the tool to calculate the scores.

**Table 1:** Readability metrics and formulae CW = complex words (three or more syllables excluding proper nouns, jargon, compound words, and common suffixes); S = sentence; Sy = syllables; W = words

Metric	Score	Description	Formulae
Gunning Fog (GF) Index	Grade level	Developed to assist American businesses in improving the readability of their writing. Applicable to a wide range of disciplines.	GF = 0.4 × [(W/S) + (CW/W) × 100]
Flesch-Kincaid Reading Grade Level	Grade level	Forms part of the Kincaid Navy personnel collection of tests. Suited to a broad range of disciplines.	FKG = [0.39 × (W/S)] + [11.8 × (Sy/W)] - 15.5
Flesch Reading Ease	Index score (0-100)	Developed to assess the readability of newspapers. Best suited for assessing school textbooks and technical manuals. Scored from 0-100, with higher scores indicating easier readability.	FRE = 206.835 - [84.6 × (Sy/W)] - [1.015 × (W/S)]
Simple Measure of Gobbledygook	Grade level	Measurement of readability based on a number of polysyllabic words. Particularly effective for health-related materials.	SMOG Grade = 3 + √(polysyllable count)

Quality analysis

The quality of the selected webpages was evaluated using the Journal of the American Medical Association (JAMA) benchmark criteria. These criteria encompass four specific domains of assessment (Table 2) [14].

**Table 2:** Journal of the American Medical Association (JAMA) benchmark criteria

Component	Description	Assessment Focus
Authorship	Transparency of author information.	Author names, affiliations, and credentials.
Attribution	Proper citation of references and copyright information.	Verification of cited sources, adherence to copyright regulations.
Disclosure	Statements revealing ownership, sponsorship, or potential conflicts of interest.	Identification of potential biases due to financial or other relationships.
Currency	Display of information, upload, and most recent update dates.	Timelines and relevance of the information, indicating how up-to-date the content is.

Statistical analysis

Statistical analysis was conducted using Stata/SE Version 18.0 (StataCorp LLC, College Station, TX). Descriptive statistics were used to calculate the mean, standard deviation, and 95% confidence intervals for each readability and quality metric. The population mean and associated confidence intervals were generated under the assumption of normally distributed data. 

The analysis was primarily descriptive in nature. No inferential statistical tests were performed, as the objective was to assess the overall readability and quality metrics across a sample of URLs, rather than to compare predefined groups. 

## Results

A total of 46 URLs were included for analysis. The literature search strategy is illustrated in Figure 1.

Reading level

The mean Flesch-Kincaid Grade Level (FKG), which estimates the US school grade level required to understand a piece of text, was 9.97 ± 3.11 (95% CI 9.04-10.89). The mean Gunning Fog Index grade level, which estimates the years of education needed to understand text, was 11.19 ± 4.16 (95% CI 9.95-12.42), and the mean FRE score, which measures how easy a text is to understand, was 43.04 ± 13.20 (95% CI 39.12-46.96). The mean SMOG grade level, estimating the number of years of education needed to understand a piece of writing, was 8.75 ± 2.88 (95% CI 7.90-9.61). Only five webpages (10.9%) met the recommended sixth-grade reading level or below on both FKG and GF scores. Eighteen webpages (39.1%) achieved an eighth-grade reading level or below according to the FKG score, while 12 webpages (26.1%) met this threshold on the GF score (Table 3).

**Table 3:** Readability metrics

Readability Metric	Mean	SD	95% Confidence Interval	Interpretation Scale
Flesch-Kincaid Grade Level	9.97	3.11	9.04-10.89	US school grade level
Gunning Fog Index	11.19	4.16	9.95-12.42	US school grade level
SMOG Index	8.75	2.88	7.90-9.61	US school grade level
Flesch Reading Ease	43.04	13.2	39.12-46.96	0-100 (higher = easier to read)

JAMA score

**Figure 2:**
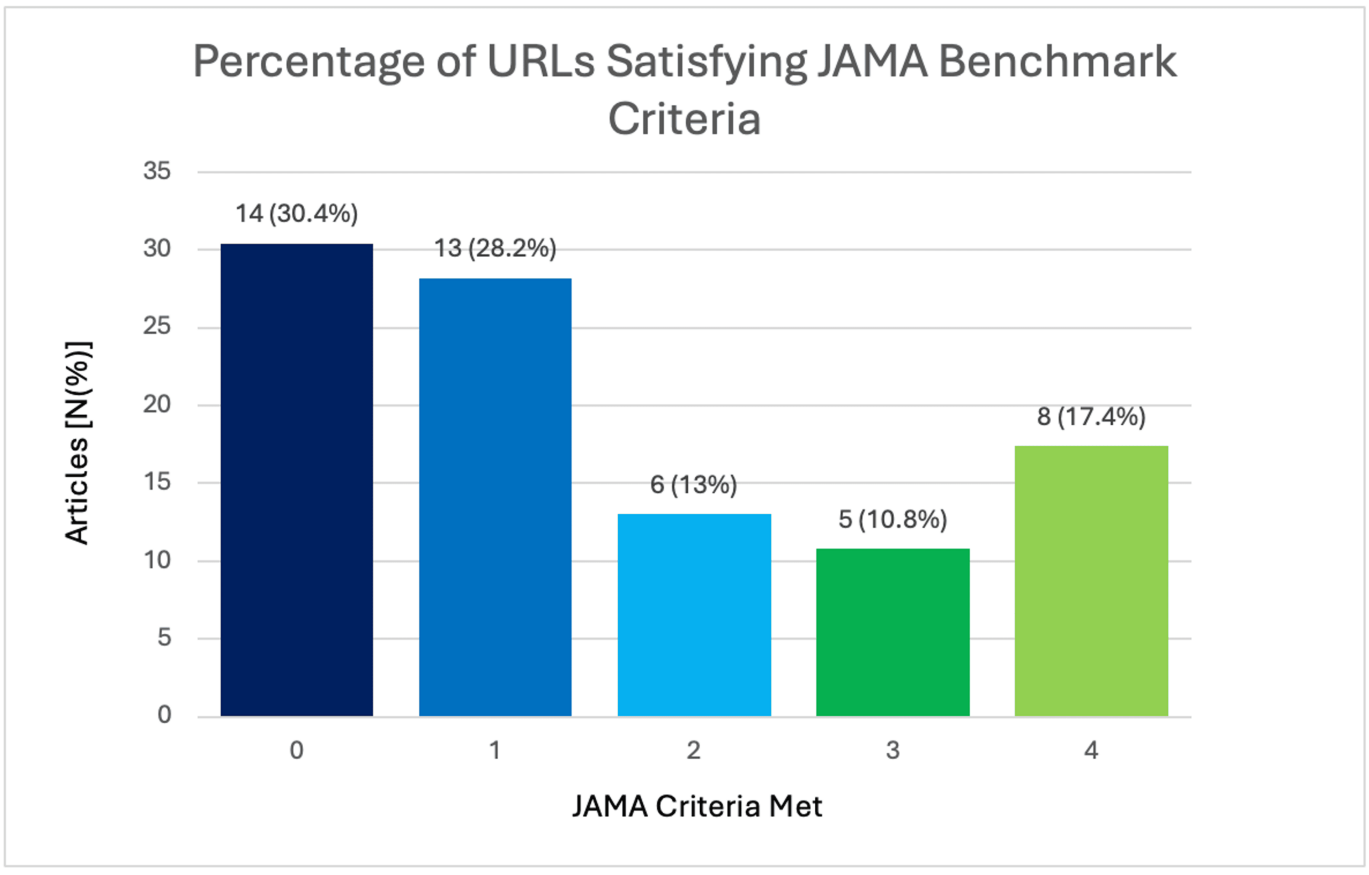
Journal of the American Medical Association (JAMA) criteria

The mean JAMA score, which is a benchmark criterion used to evaluate the quality and readability of online health information, across all webpages was 1.43 ± 1.46 (95% CI 1.00-1.87). Authorship was disclosed in 26 webpages (56.5%), attribution was present in 15 webpages (32.6%), upload dates were provided in 23 webpages (50%), and relevant disclosures were made in eight webpages (17.4%). In total, 14 webpages (30.4%) scored 0 on the JAMA criteria, 13 webpages (28.3%) scored 1, six webpages (13.0%) scored 2, five webpages (10.1%) scored 3, and eight webpages (17.4%) scored 4 (Figure 2).

## Discussion

Our analysis confirms that most online materials related to osteochondral knee injuries are written above the recommended readability levels and are difficult to read, and also lack markers of quality. This reduces the ability of such materials to properly inform and prepare patients for certain surgical procedures. Providing thorough preoperative education is crucial when treating osteochondral knee injuries, as it helps manage patient expectations and supports proper adherence to post-operative rehabilitation protocols, which both play a critical role in overall patient outcomes [1-4].

These findings are consistent with previous research on other orthopaedic procedures. Notably, a recently performed similar study evaluating online resources for total hip arthroplasty (THA) also demonstrated that the majority of online content exceeded recommended readability levels and fell short in quality indicators [15]. In that study, the mean FKG was 9.5 (vs. 9.97 in the present study), the Gunning Fog Index was 11.1 (vs. 11.19), and the FRE score was slightly higher at 48.5 (vs. 43.04). The JAMA benchmark score was also comparable at 1.4 (vs. 1.43). Despite evaluating a different orthopaedic topic, both studies highlight a persistent issue, that online health content related to orthopaedic surgery remains difficult to comprehend and inconsistently meets quality standards. These findings reinforce the need for systemic improvements in how digital patient education is created and delivered.

Clinician-patient communication is vital in orthopaedic surgery, yet prior studies would suggest that patients often receive less information than desired [16]. A consequence is that patients would often seek additional information from alternative sources, like the internet. In recent years, the internet has provided open access to medical information, enabling patients to independently research complicated medical topics. However, without standardised oversight, misleading or low-quality information can negatively affect clinician-patient trust and affect clinical outcomes [17,18].

The American Medical Association (AMA) and the National Institutes of Health (NIH) have recommended that patient information be accessible at a sixth-grade reading level [19,20]. Our results indicate that most online content related to osteochondral injuries of the knee falls short of this metric. This particular issue is not unique to orthopaedic surgery, with very similar findings observed across most studies assessing the readability of online health-related content across various medical and surgical specialities [21,22]. There should be a need to address this mismatch, as lower levels of health literacy have been linked to poorer adherence to the recommended postoperative care plans, affecting overall recovery and patient outcomes [23]. This mismatch between available health content and patient reading levels must be addressed, as lower health literacy has been linked to poorer adherence to postoperative care. Clear, standardised readability practices and collaborative efforts are necessary to close this gap.

Healthcare-related websites should prioritise creating patient-centred content for all medical conditions or operative procedures at an appropriate level. Osteochondral injuries of the knee are a complicated topic, and medical information available online, delivered at an inappropriate level, can further exacerbate this misunderstanding for patients. Websites could aim to improve their content delivery by means of simplifying the language being used, incorporating visual aids, and regularly reviewing posted content to ensure it corresponds with health literacy standards [24]. Websites could incorporate a “Clinicians” and “Patients” option to provide relevant information to the relevant reader. The collaboration between orthopaedic specialists, other surgical specialities, and health literacy experts could encourage the production of clearer, more understandable, and effective online educational materials. Interactive content, such as frequently asked questions (FAQs) and decision-making tools, can improve patient understanding and engagement by offering accessible, personalised support formats [18]. The findings of this study are consistent with previous research on the readability of online medical information in orthopaedic surgery. Prior studies frequently report that health-related content online exceeds the recommended reading levels, which typically require an 8th-grade or higher reading level [8,9,15,25]. A recent systematic review determined that online patient information regarding common sports injuries did not reach the readability recommendations set out by the AMA and NIH, with 11 studies being evaluated, which revealed an FKG level of 10.5, which far exceeds the sixth-grade reading level recommended [8].

Official trusted government and institutional websites also face similar issues. A study evaluating COVID-19 across 15 countries found that all assessed materials exceeded the recommended reading levels, with median FKGs ranging from 9.4 to 13.1, well above recommendations [25]. This highlights the broader need for improving digital health content accessibility, even among reputable sources [25].

Our study found significant variability in the quality of online health information related to osteochondral injuries of the knee, with only eight websites (17.4%) achieving the top JAMA score of 4. Similar studies in orthopaedic surgery indicate significant concerns regarding the quality of online patient information, though the distribution of scores may vary slightly [26]. Fourteen of the URLs evaluated (30.4%) scored 0 on the JAMA scale, indicating no adherence to the assessed set quality standards, which strongly suggests more work is required to bring online material related to osteochondral injuries of the knee up to the desired set standard [9]. Authorship was disclosed in 26 websites (56.5%), compared to approximately 45% in similar orthopaedic studies [27]. Authorship is an essential assessment criterion as it provides transparency regarding content credibility and accountability [14]. Attribution of information was only found on 15 of the assessed resources (32.6%), which is consistent with findings from other orthopaedic health-related content focusing on hip and spinal procedures, which shows consistent limited citation practices. The currency of information was found in 23 of the websites (50%), corresponding with results from other orthopaedic studies evaluating online patient education material [26,27]. However, disclosure of ownership or potential conflicts of interest was notably limited, with only eight websites (17.4%) including such information [28]. These deficits in transparency diminish trustworthiness and hinder effective patient education.

Limitations

This study has several limitations that should be acknowledged. The search terms used were based on their clinical relevance to the topic under investigation. However, these search terms may not reflect the language used by patients when searching for health information online. Different search terms may have yielded different results. As such, the search strategy may have introduced section bias towards more medically technical websites. Search engines make use of algorithms, which are personalised to the individual, making replication difficult. Our analysis was restricted to English-language websites, which may limit the generalizability of our findings. It should be acknowledged that patients with limited English may be at even greater risk of setbacks in accessing and understanding relevant health information. Our evaluation focused on the readability and credibility of the source of the information, rather than the medical accuracy of the content being presented. This study primarily assessed online resources and did not include local hospital-based patient education materials and content, such as pre-operative information booklets or rehabilitation guidelines and instructions, which are often extensively reviewed for readability, accuracy, and ethical measures set by hospital committees, prior to their acceptance and distribution to the patients that are being treated. The exclusion of these resources limits the generalizability of the findings, particularly regarding the comprehension of patient education materials provided in clinical hospital-based settings. Additionally, the readability scores were generated using automated calculations, which, while widely used in similar prior research studies, may not fully account for medical jargon, the complexity of sentence structure, or the contextual meaning of the content. Future studies could consider comparing hospital-based patient education materials with online information, performing content validation analyses, and assessing the influence of visual aids and interactive components on patient comprehension.

## Conclusions

This study highlights significant concerns regarding the readability and quality of online patient education materials related to osteochondral injuries of the knee. Our findings indicate that most publicly accessible healthcare content exceeds recommended readability levels, making it challenging for the average patient to understand. However, it is important to interpret these findings in the context of several limitations. This study focused specifically on the readability and source credibility of healthcare content available to patients but did not assess the clinical accuracy of the content. The exclusion of hospital-based patient education materials means that our analysis reflects only publicly accessible online information, instead of all available patient education resources. Despite the limitations discussed, our study re-emphasises the obvious need for improved accessibility and reliability of online healthcare content. Given the variability and complexity of online content, clinicians should supplement patient education with clear, personalised guidance to help reduce confusion and ensure understanding. Future research should focus directly on comparisons between hospital-provided educational materials and general online materials, as well as evaluate the clinical accuracy of information, in conjunction with readability metrics. While universal enforcement of readability and quality standards across online health content is unlikely, healthcare professionals and institutions alike play a critical role in guiding patients to reputable sources. This can help fill the gap and promote high-quality, patient-centred educational material being advocated for best medical and surgical practices.

## Appendices

**Table 4:** List of URLs included in analysis URL = uniform resource locator

	URLs
1	https://stanfordhealthcare.org/medical-conditions/bones-joints-and-muscles/chondral-osteochondral-defect.html
2	https://rileywilliamsmd.com/osteochondral-defect-articular-cartilage-damage-manhattan-new-york-city-ny/
3	https://cathaljmoran.com/osteochondral-cartilage-defect/
4	https://radiopaedia.org/articles/osteochondral-defect
5	https://www.orthobullets.com/knee-and-sports/3133/articular-cartilage-defects-of-knee
6	https://www.cedars-sinai.org/health-library/diseases-and-conditions/o/osteochondral-lesionsosteochondritis-dessicans.html
7	https://www.domontortho.com/osteochondral-defect-knee-orthopedic-surgeon-lincolnshire-libertyville-il.html
8	https://davidslattery.com/knee-conditions/ocd/
9	https://www.physio-pedia.com/Articular_Cartilage_Lesions_of_the_Knee
10	https://thefootballphysio.co.uk/osteochondral-defects-of-the-knee/
11	https://www.orthobullets.com/knee-and-sports/3028/osteochondritis-dissecans
12	https://www.iskinstitute.com/kc/knee/osteochondral_defect/osteochondral_defect.html
13	https://pmc.ncbi.nlm.nih.gov/articles/PMC3535119/
14	https://www.arthroscopytechniques.org/article/S2212-6287(22)00068-8/fulltext
15	https://www.sciencedirect.com/science/article/pii/S2667254524000088
16	https://www.physio-pedia.com/Osteochondritis_Dissecans_of_the_Knee
17	https://www.kneesurgeonbristol.co.uk/conditions-treatments/joint-surface-damage/
18	https://www.wheelessonline.com/joints/knee/chondral-and-osteochondral-injuries-of-the-knee/
19	https://wikism.org/Osteochondral_Defect_Knee
20	https://bmcmusculoskeletdisord.biomedcentral.com/articles/10.1186/s12891-020-03531-8
21	https://aoj.amegroups.org/article/view/4405/html
22	https://os.clinic/conditions/foot-ankle/osteochondral-injuries/
23	https://pmc.ncbi.nlm.nih.gov/articles/PMC9222836/
24	https://www.physio.co.uk/what-we-treat/surgery/knee/micro-fracture-of-an-osteochondral-lesion.php
25	https://www.england.nhs.uk/wp-content/uploads/2022/10/2007-oca-spec-comm-policy.pdf
26	https://radiologyinplainenglish.com/osteochondral-lesion/
27	https://oxfordortho.sg/conditions/general/osteochondral-injuries/
28	https://gustavelorthopedics.com/Osteoarthritis-and-Osteochondral-Injuries
29	https://www.sportsmedtoday.com/osteochondral-lesion-va-218.htm
30	https://www.frontiersin.org/journals/cell-and-developmental-biology/articles/10.3389/fcell.2020.580868/full
31	https://www.kneeman.com/chondral-injuries-of-the-knee-orthopaedic-surgeon-fairfax-woodbridge-va.html
32	https://www.keithschroedermd.com/understanding-osteochondral-defects-causes-symptoms-and-treatment-options/
33	https://pubs.rsna.org/doi/abs/10.1148/rg.2018180044?journalCode=radiographics
34	https://www.thesteadmanclinic.com/patient-education/knee/chondral-defects
35	https://www.epainassist.com/joint-pain/knee-pain/what-is-osteochondral-defect
36	https://www.allfitwell.com/osteochondral-defect/
37	https://www.jospt.org/doi/full/10.2519/jospt.2014.5140
38	https://www.orthopaedia.com/chondral-injuries-of-the-knee/
39	https://www.massgeneral.org/assets/mgh/pdf/orthopaedics/sports-medicine/physical-therapy/rehabilitation-protocol-for-osteochondral-autograft-allograft-transfer-system-oats-procedure.pdf
40	https://www.hcahealthcare.co.uk/conditions/osteochondral-defects
41	https://www.iipseries.org/assets/docupload/rsl2024BE10045EBCA566B.pdf
42	https://www.jospt.org/doi/10.2519/jospt.2012.3673
43	https://www.hipandkneemanchester.co.uk/osteochondral-injuries-specialist-manchester-cheshire.html
44	https://www.joionline.net/library/osteochondral-fracture/
45	https://pmc.ncbi.nlm.nih.gov/articles/PMC7930143/
46	https://pmc.ncbi.nlm.nih.gov/articles/PMC7673409/
